# Contribution of ERMES subunits to mature peroxisome abundance

**DOI:** 10.1371/journal.pone.0214287

**Published:** 2019-03-25

**Authors:** Michela Esposito, Sylvie Hermann-Le Denmat, Agnès Delahodde

**Affiliations:** 1 Institute for Integrative Biology of the Cell (I2BC), CEA, CNRS, Univ. Paris‐Sud, Université Paris‐Saclay, Gif‐sur‐Yvette cedex, France; 2 Ecole Normale Supérieure, PSL Research University, Paris, France; University of Parma, ITALY

## Abstract

Eukaryotic organelles share different components and establish physical contacts to communicate throughout the cell. One of the best-recognized examples of such interplay is the metabolic cooperation and crosstalk between mitochondria and peroxisomes, both organelles being functionally and physically connected and linked to the endoplasmic reticulum (ER). In *Saccharomyces cerevisiae*, mitochondria are linked to the ER by the ERMES complex that facilitates inter-organelle calcium and phospholipid exchanges. Recently, peroxisome-mitochondria contact sites (PerMit) have been reported and among Permit tethers, one component of the ERMES complex (Mdm34) was shown to interact with the peroxin Pex11, suggesting that the ERMES complex or part of it may be involved in two membrane contact sites (ER-mitochondria and peroxisome- mitochondria). This opens the possibility of exchanges between these three membrane compartments. Here, we investigated in details the role of each ERMES subunit on peroxisome abundance. First, we confirmed previous studies from other groups showing that absence of Mdm10 or Mdm12 leads to an increased number of mature peroxisomes. Secondly, we showed that this is not simply due to respiratory function defect, mitochondrial DNA (mtDNA) loss or mitochondrial network alteration. Finally, we present evidence that the contribution of ERMES subunits Mdm10 and Mdm12 to peroxisome number involves two different mechanisms.

## Introduction

The biological importance of links between organelles is well recognized and there is growing evidence that mitochondria and peroxisomes exhibit closer interrelationships than previously appreciated [1,2]. These connections include metabolic cooperation, crosstalk and physical contact sites (PerMit) [3–5]. Moreover, mitochondria and peroxisomes share key components of their division machinery [6,7]. The endoplasmic reticulum (ER) plays also an essential role in peroxisomal and mitochondrial biogenesis. Peroxisomes can originate from the ER and mitochondria communicate with the ER through contact sites. Altogether, these connections are key players in cell signaling, lipid homeostasis and maintenance of organelle morphology.

Mitochondria are indispensable organelles for eukaryotic cell function and are transferred from mother to daughter cells during cell division because mitochondria cannot be synthesized *de novo*. They are connected to the ER through a specialized protein complex called the Endoplasmic Reticulum-Mitochondria Encounter Structure (ERMES complex, [8]). ERMES comprises four proteins: the mitochondrial outer membrane protein Mdm10, the ER-resident Mmm1 protein and two peripheral membrane proteins, Mdm34 and Mdm12. All four proteins are required for complex formation. When any one of ERMES subunits is missing, other subunits fail to localize at the contact sites [8]. With the exception of Mdm10, ERMES proteins contain a SMP domain (synaptotagmin-like mitochondrial-lipid-binding domain) belonging to the TULIP superfamily domain of an important group of proteins that bind lipids and other hydrophobic ligands within a central, tubular cavity [9]. ERMES proteins have also been implicated in mitochondrial functions including morphology [10–13], mitochondrial protein import [14], genome maintenance (ERMES has been shown to co-localize with actively replicating mitochondrial nucleoids) [13,15–17], and mitophagy [18]. The Mdm10 subunit is also a component of the sorting and assembly machinery (SAM) that allows the assembly of membrane β-barrel proteins in the mitochondrial outer membrane [14]. The ERMES complex plays also a role in the association between mitochondria and the actin cytoskeleton with Mmm1, Mdm10 and Mdm12 being essential for actin-dependent mitochondrial motility [19]. The mitochondrial calcium-binding GTPase Gem1 has been identified as a new ERMES subunit supposed to be an integral regulatory component of ERMES [20,21]. Given the lipid exchange between the ER and the mitochondrial outer membrane and the presence of SMP domains in three ERMES components, it has been suggested that ERMES plays an important role for efficient inter-organelle phospholipid and calcium exchange. Based on electron microscopy and x-ray structure analyses, a model proposes that a tubular-shaped, hydrophobic, hetero-tetrameric channel can be formed by the SMP domains of Mmm1 and Mdm12 to transport lipids [22,23]. Additionally, lipid transfer assays were recently used to demonstrate that the Mmm1-Mdm12 complex exerts efficient phospholipid transfer between membranes *in vitro* [24]. Even though numerous functions have been associated with the ERMES complex and/or its constituents, ERMES deficiency is not lethal due to the existence of redundant pathways. As an example, ERMES function can be bypassed by the activity of other contact sites such as mitochondria-vacuole contacts [25–27].

Peroxisomes are ubiquitous multifunctional organelles that carry out the β-oxidation of fatty acids and the neutralization of hydrogen peroxide. They are limited by a single membrane and are devoid of genetic material. Peroxisomes are remarkably dynamic, responding to environmental and cellular signals by alterations in size, number and proteomic content. They can either be formed *de novo* from the ER or by the fission of pre-existing peroxisomes [28–31]. The metabolic cooperation and crosstalk of mitochondria and peroxisomes render them dependent upon each other for their proper function. It has been shown that similar nutritional stimuli increase biogenesis of both organelle [32]. A loss of mitochondrial respiratory function can promote peroxisome biogenesis [33–35] and mitochondrial dysfunctions are observed under conditions in which peroxisome biogenesis is impaired [36]. Altogether these findings strongly suggest a coordination of the peroxisomal and mitochondrial biogenesis.

In yeast, independent studies have identified ERMES subunits as potential players in establishing contact sites between mitochondria and peroxisomes. Mdm10 has been thus identified in systematic analyses of the effects of gene deletion on peroxisome biogenesis or the utilization of fatty acids [3,37,38] and, Mdm10, Mdm12 and Mdm34 were identified as affecting the localization of Pex11, a peroxin involved in peroxisome proliferation [4]. It was further showed that Pex11 interacts physically with the mitochondrial Mdm34 protein suggesting a potential role of Pex11 in establishing a contact site between peroxisomes and mitochondria via the ERMES complex or a part of it [4]. As a result, it can be hypothesized that ERMES components play a regulatory role in establishing contacts between two different organelles depending on cellular needs.

In the present study, we deciphered the involvement of each ERMES component on mature peroxisome abundance. As previously described [3,4], we found that cells lacking Mdm10 or Mdm12 exhibit a large increase in the number of mature peroxisomes compared to wild-type cells. On the contrary, cells lacking Mmm1 or Mdm34 exhibit wild-type level of peroxisomes. We show that the mitochondria and peroxisome defects due to the absence of Mdm10 or Mdm12 can be dissociated. We also present evidence that the high peroxisome abundance generated in absence of Mdm12 or Mdm10 probably arises by different mechanisms.

## Results

### Cells deleted for MDM10 or MDM12 contain an increased number of mature peroxisomes

**Fig 1:**
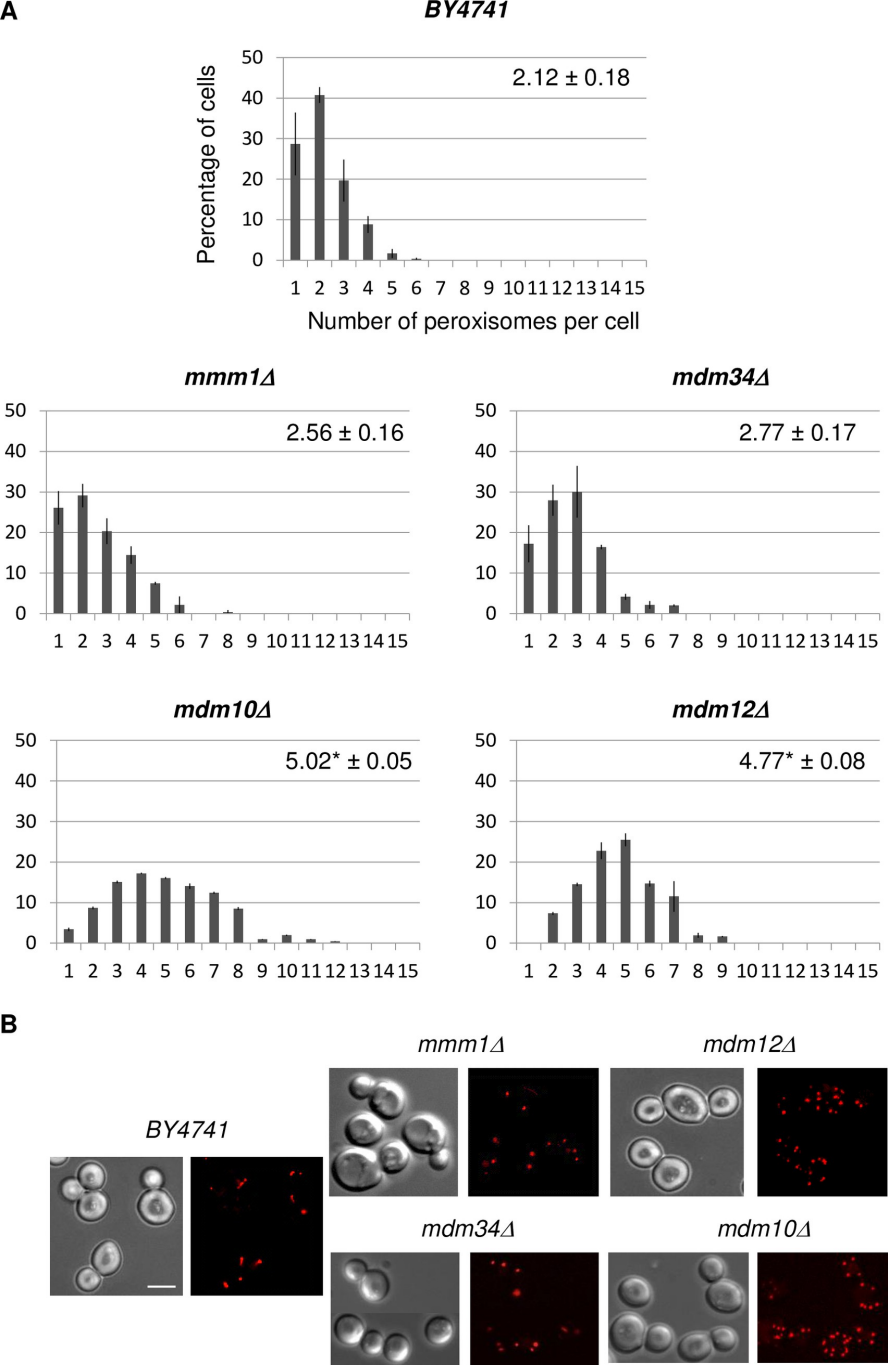
Different contribution of ERMES components to peroxisome population. (A) Percentage of cells for a given number of peroxisomes per cell is shown for the wild type and the four ERMES mutant strains grown on glucose. For each strain, the number of peroxisomes per cell was counted from images of two counts of at least 100 non-budding cells from three independent experiments. Bars represent the standard deviation (SD). On each graph, the average number (± SD) of peroxisomes is indicated. (*) indicates that the difference between a mutant and the reference strain (BY4741) is significant after statistical analyses (Student’s *t* test, *p-values* < 0.005). (B) Wild-type and ERMES mutant cells expressing a Ds.Red protein targeted to the peroxisomal matrix (Ds.Red-SKL) were analyzed by fluorescence microscopy. Typical views of the five different strains are shown (scale bar 5 μm).

Three of the four ERMES components (Mdm10, Mdm12 and Mdm34) were identified in a high throughput imaging-based screen designed to uncover proteins that play a role on peroxisome biogenesis in yeast [3]. There, Mdm10, Mdm12 or Mdm34 absence resulted in aberrant (usually several small) peroxisomes in cells grown to stationary phase. Beside to the phase of growth, peroxisomes also respond to environmental and cellular signals by alterations in size and number. They have notably been shown to multiply in response to mitochondrial dysfunction, a hallmark of ERMES mutants [33–35]. We therefore re-examined the number of mature peroxisomes in ERMES deletion strains (*mmm1Δ*, *mdm34Δ*, *mdm10Δ*, *mdm12Δ*) in exponentially growing cells under condition of constitutive peroxisome division (glucose) and under condition that induces peroxisome proliferation (oleic acid). All strains examined were isogenic to the BY4741 strain and expressed the red fluorescent protein (Ds.Red) fused to the peroxisomal targeting signal PTS1 (Ds.Red-SKL) to mark mature peroxisomes capable of importing matrix proteins. Quantitative distribution of peroxisomes (Fig 1A and Table 1) indicates that deletion of *MDM10* or *MDM12* led to a two-fold increase in peroxisome numbers per cell grown in glucose and observed at late exponential phase (see Materials and Methods). Equivalent results were obtained at early exponential phase (data not shown). On the contrary, deletion of *MMM1* or *MDM34* did not significantly change the abundance of peroxisomes compared to the wild-type strain. Oleate was able to further induce peroxisome proliferation in all deleted strains, even though *mdm10Δ* cells showed a moderate increase in peroxisome number in that particular growth condition (Table 1). Similar results were obtained for *mmm1Δ*, *mdm34Δ*, *mdm10Δ and mdm12Δ* spores issued from the cross of haploid BY4741 mutants and the wild-type haploid W303-1B strain (Table 1). Importantly, expression of a wild-type *MDM12* allele in the *mdm12Δ* mutant strain re-established a wild-type level of peroxisomes in the functional complemented strain (Table 1).

**Table 1:** Average numbers (± SD) of peroxisomes in ERMES mutant strains in two different genetic backgrounds.

strain	Mean ± SD	
	Glucose	Oleate
BY4741	2.12 ± 0.18	3.84 ± 0.09
*mmm1Δ*	2.56 ± 0.16	4.81 ± 0.88
*mdm34Δ*	2.77 ± 0.17	6.23 ± 0.42
*mdm10Δ*	5.02* ± 0.05	6.01 ± 0.01
*mdm12Δ*	4.77* ± 0.08	6.88* ± 0.19
BY/W303	2.31 ± 0.14	4.0 ± 0.41
*mmm1Δ*	2.33 ± 0.27	4.59 ± 0.59
*mdm34Δ*	2.82 ± 0.45	6.39 ± 0.18
*mdm10Δ*	5.32* ± 0.4	7.44* ± 0.14
*mdm12Δ*	5.12* ± 0.01	9.53* ± 0.4
WT + *MDM12*	2.2 ± 0.3	4.25 ± 0.3
*mdm12Δ* + *MDM12*	2.6 ± 0.2	4.65 ± 0.1

Average numbers of peroxisomes per cell observed in the different ERMES mutant strains (BY4741 and mixed BY/W303 genetic backgrounds) grown in glucose or oleate and, presented as mean ± SD (Standard Deviation). Statistical analysis (Student’s test) revealed that the differences in average number of peroxisomes in *mdm10Δ* and *mdm12Δ* cells, but not in *mmm1Δ* and *mdm34Δ* cells, is significantly different compared to the wild-type controls (*, *p-values* < 0.005). For each strain, the number of peroxisomes was counted from images of two counts of at least 100 non-budding cells from three independent experiments.

Because the cellular abundance of peroxisomes has been shown to increase in response to mitochondrial dysfunction [33–35] and ERMES mutants are well-known to lose their mitochondrial genome, we next examined mitochondrial morphology, respiratory competence and presence of mtDNA in all ERMES deletion strains examined for peroxisome content. Mitochondria of the ERMES deletion strains were visualized by the detection of a mitochondrial targeted GFP (mtGFP) and presence of mitochondrial DNA (mtDNA) by DAPI staining or detection of the mtDNA-binding protein Abf2 fused to GFP (S1 Fig). Abf2 is a major component of mitochondrial nucleoid, the nucleoprotein complex where mtDNA is compacted. All ERMES mutant strains of the mixed nuclear background (BY4741/W303) were devoid of mtDNA (rho0 state) and unable to grow on glycerol, a non-fermentable substrate, whereas the isogenic BY4741 *mdm34Δ* and *mdm12Δ* strains were able to grow, while poorly, on glycerol (S1 Fig). In both genetic contexts, the mutant cells exhibited abnormal mitochondrial structures (S1 Fig). To exclude that the rho0 state could by itself participate in abnormal peroxisome abundance, peroxisome content was analyzed in a rho+ and a rho0 BY4741 strain (S2 Fig). In this wild-type reference context, the rho0 state had no significant effect on peroxisome abundance. Hence, the abnormal peroxisome population observed in *mdm10Δ* and *mdm12Δ* cells is not simply due to a loss of the mitochondrial genome, loss of respiratory function or morphologic alteration of the mitochondrial network. Altogether, our results show that absence of only two (Mdm10 and Mdm12) of the four proteins forming the ERMES complex, leads to an increase abundance of cellular peroxisomes in exponentially growing cells.

### Dominant point mutations in *VPS13* do not restore wild-type level of peroxisomes in *mdm10*Δ and *mdm12*Δ cells

**Fig 2:**
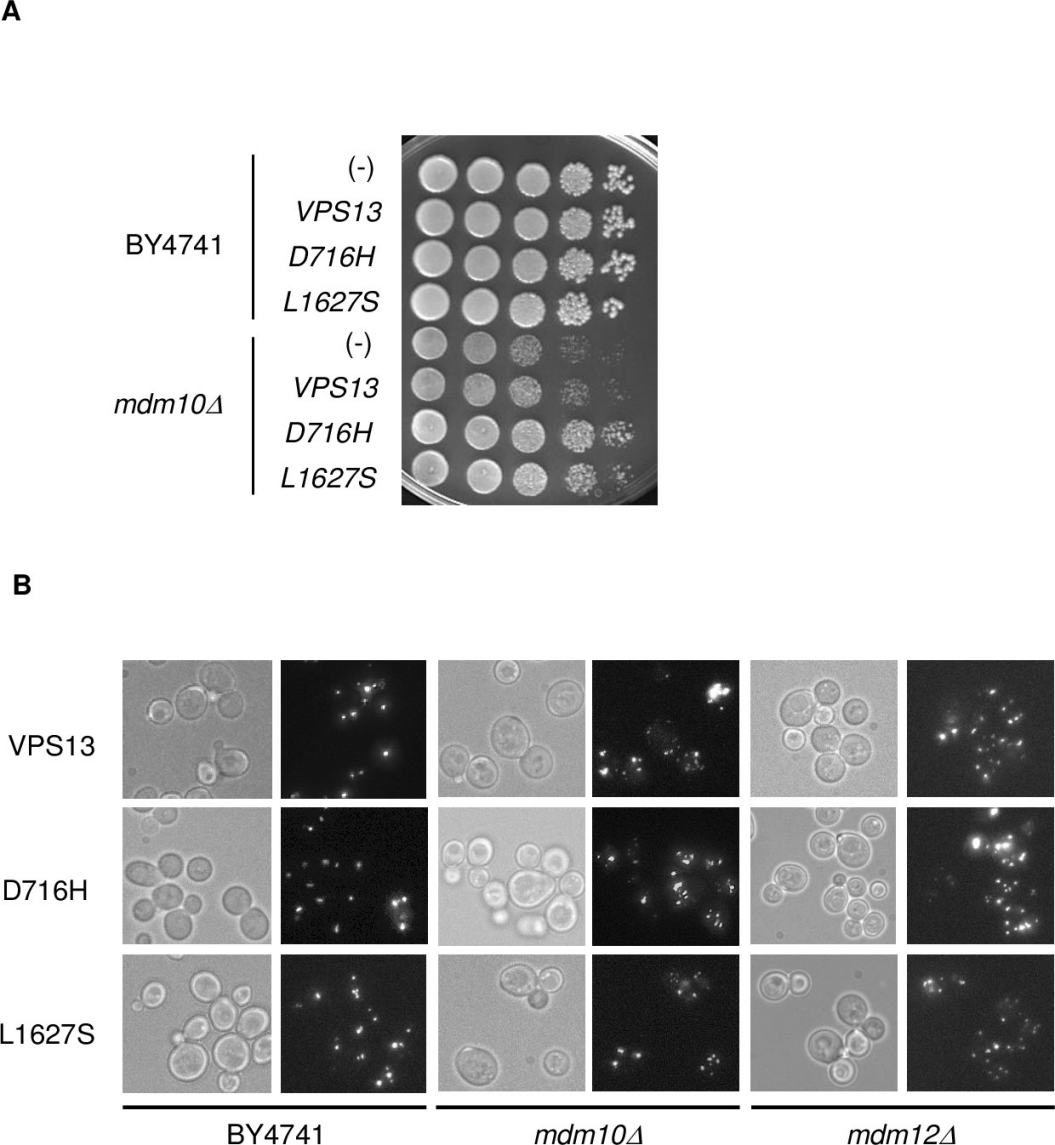
Dominant mutations in *VPS13* do not alleviate the *mdm10Δ* and *mdm12Δ* peroxisome defect. (A) Wild-type and *mdm10Δ* cells were transformed by either an empty plasmid (-) or a plasmid bearing the wild-type *VPS13* gene (*VPS13*), the (D716H) mutant allele or the (L1627S) mutant allele. Transformants were spotted on YPD after serial dilutions and incubated at 28°C for 3 days. (B) Wild-type, *mdm10Δ* and *mdm12Δ* cells transformed with the plasmid expressing *VPS13* or D716H or L1627S were further transformed by a plasmid expressing GFP targeted to the peroxisomal matrix (GFP-SKL) to mark mature peroxisomes. Transformants were analyzed by fluorescence microscopy. Typical views of the different strains are shown.

The absence of lethality associated to ERMES deficiency is due to the existence of redundant pathway and mitochondrial phenotypes associated with ERMES mutants can indeed be bypassed by dominant point mutations in the endosomal protein-encoding gene *VPS13* (vacuolar protein sorting 13; [25,39]). More precisely, the single amino-acid substitutions D716H and L1627 were identified as suppressing the growth defects associated to *mmm1Δ* cells, *mdm10Δ* cells, and *mmm1-1* temperature-sensitive mutant cells [25,39]. The D716H suppressor allele was also found to restore a tubular mitochondrial network and mtDNA stability in *mmm1Δ* cells but not the assembly of the ERMES complex. Importantly, the sequence of the chromosomal *VPS13* gene has been checked in the wild-type BY4741, *mdm10Δ* and *mdm12Δ* strains and none dominant mutations was present (data not shown). To evaluate whether *VPS13* dominant suppressor alleles (hereafter named D716H and L1627S) could rescue the peroxisome defect of *mdm10Δ* and *mdm12Δ* cells, those strains and the wild-type strain were transformed with a low copy plasmid bearing the wild-type *VPS13* gene or the D716H and L1627S dominant alleles and peroxisomes were visualized by the detection of a peroxisome targeted GFP (GFP-SKL; Fig 2). As previously observed [25], expression of both *VPS13* suppressor alleles improved *mdm10Δ* growth on glucose (Fig 2A). However, D716H and L1627S expression did not re-establish a wild-type level of peroxisomes in *mdm10Δ* or *mdm12Δ* cells (Fig 2B). Hence, while dominant mutations in *VPS13* can restore growth of ERMES mutants, their presence has no impact on the peroxisome abundance defect we observed for *mdm10Δ* or *mdm12Δ* cells, indicating that mitochondria and peroxisome defects associated to ERMES mutants can be dissociated.

### Overexpression of *MCP1* or *MCP2* do not re-establish wild-type level of peroxisomes in *mdm10*Δ and *mdm12*Δ cells

**Fig 3:**
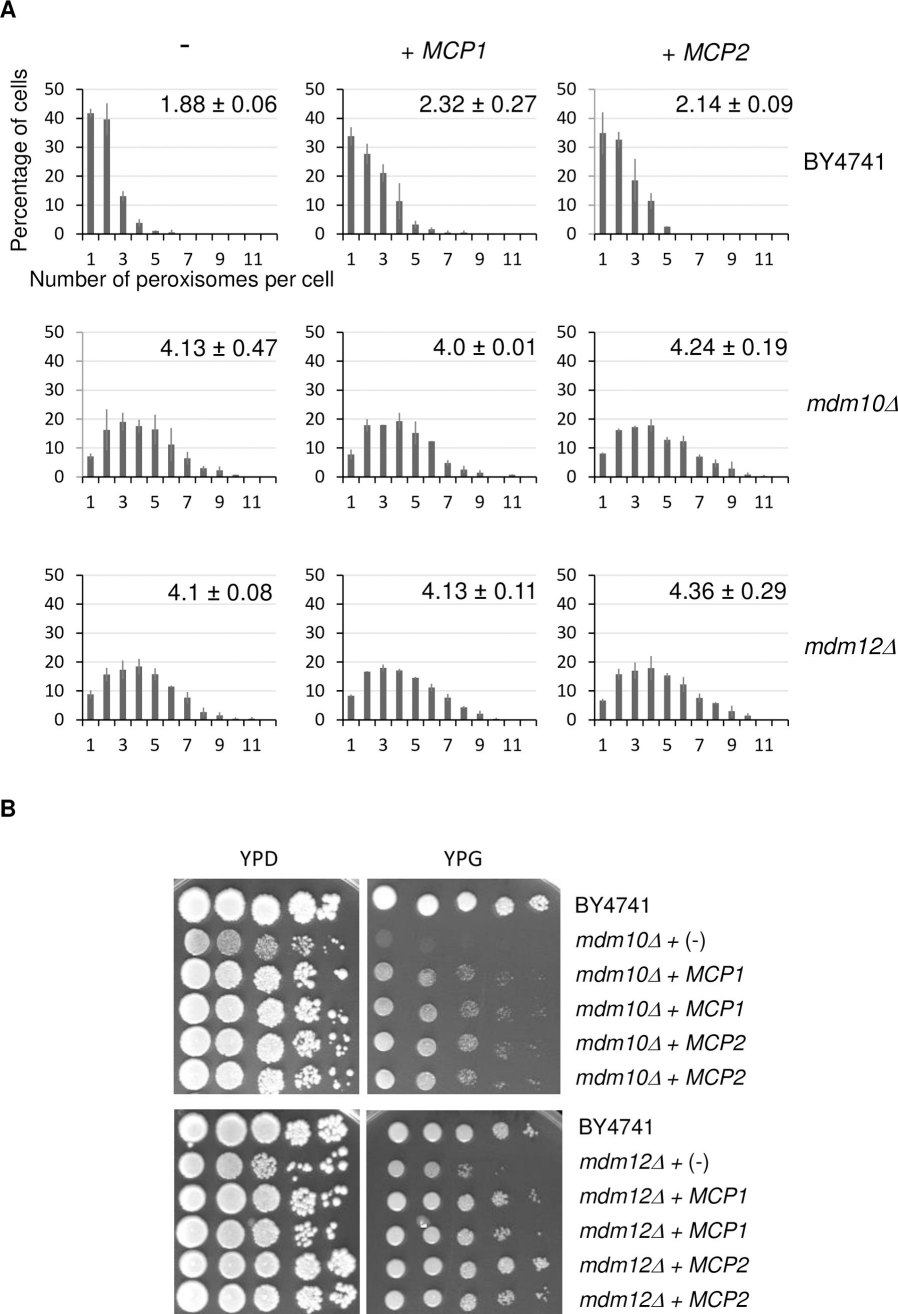
Mcp1 and Mcp2 do not suppress the *mdm10Δ* and *mdm12Δ* peroxisome defect. (A) Percentage of cells for a given number of peroxisomes per cell is shown for the wild type, *mdm10Δ* and *mdm12Δ* cells overexpressing either *MCP1* or *MCP2* or transformed with an empty plasmid (-). Cells were grown on glucose. For each strain, the number of peroxisomes per cell was counted from images of two counts of at least 100 non-budding cells from two independent experiments. Bars represent the SD. On each graph, the average number (± SD) of peroxisomes is indicated. (B) *MCP1* and *MCP2* restore *mdm10Δ* and *mdm12Δ* growth defect on glycerol. Cells of the indicated deletion strains were transformed with a plasmid encoding Mcp1 or Mcp2 or an empty plasmid (-). Growth was analyzed by drop dilution assay on YPD and YPG medium incubated at 28°C for three and five days, respectively. Two different transformants were tested for *mdm10Δ* and *mdm12Δ* cells overexpressing the *MCP1* or *MCP2* gene.

*MCP1* and *MCP2* are two other genes whose overexpression was found to suppress the growth defects of strains deleted for *MMM1*, *MDM10*, *MDM12* or *MDM34* [40]. *MCP1* and *MCP2* encode mitochondrial proteins involved in mitochondria morphology but of unknown function. Their overexpression also restored the stability of respiratory chain complexes of mitochondria devoid of *MDM10* but was unable to rescue the defect in assembly of MOM proteins observed in these cells [40]. In order to determine whether overexpression of *MCP1* or *MCP2* can restore peroxisome alterations in *mdm10Δ* and *mdm12Δ* strains, high copy number plasmid encoding Mcp1 or Mcp2 were introduced in those strains and cellular peroxisome population analyzed (Fig 3A). Overproduction of Mcp1 or Mcp2 in the wild-type strain had no significant effect on peroxisome abundance. Despite rescuing growth defect of *mdm10Δ* and *mdm12Δ* strains on glycerol, *MCP1* and *MCP2* overexpression did not modify the abnormal peroxisome number observed in these strains (Fig 3B).

### Pex11 contributes to high peroxisome proliferation in *mdm12*Δ cells

**Fig 4:**
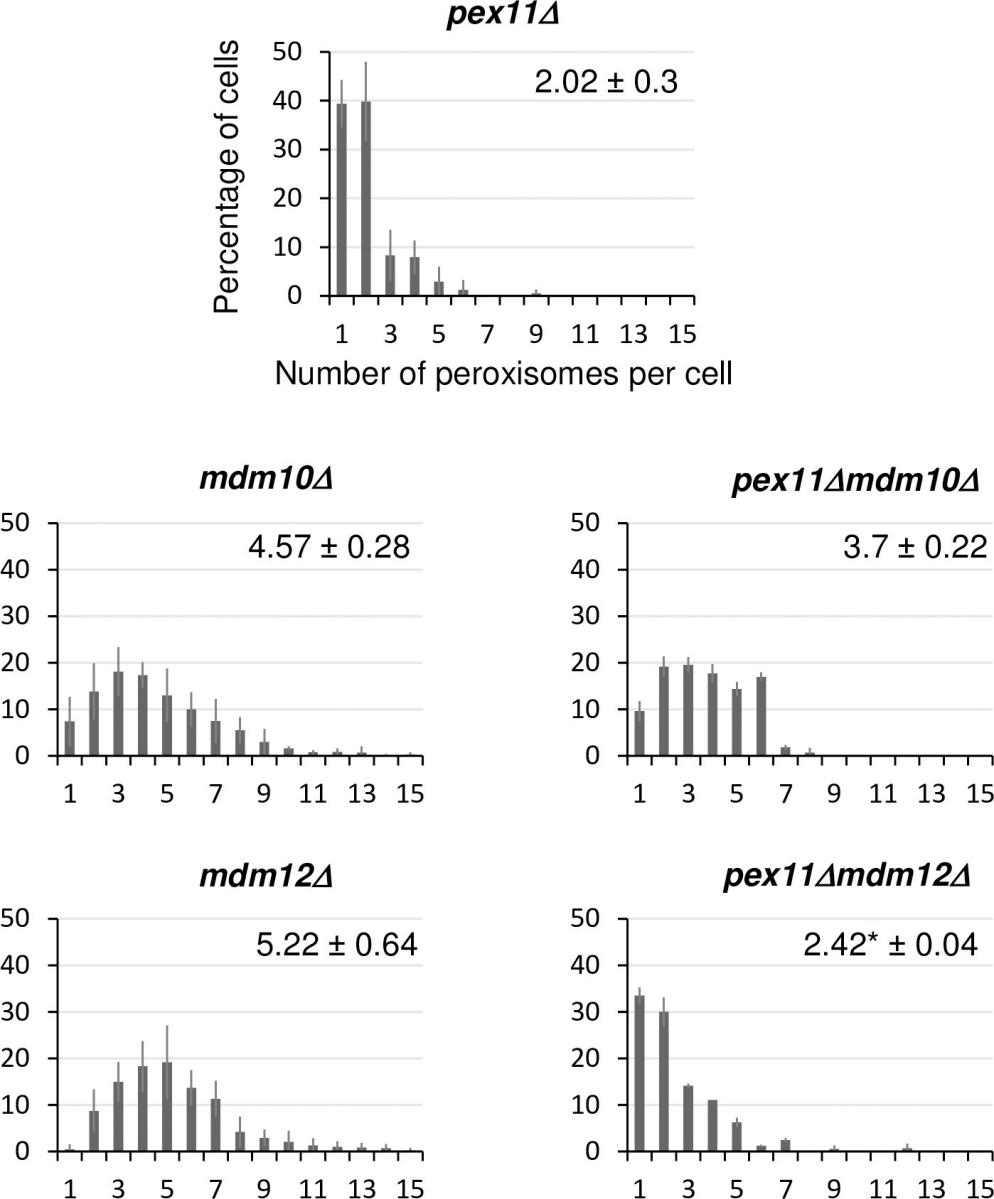
Deletion of *PEX11* re-establishes a wild-type peroxisome level in *mdm12Δ* cells. Percentage of cells for a given number of peroxisomes per cell is shown for the single *pex11Δ*, *mdm10Δ* and *mdm12Δ* mutants and for the *pex11Δmdm10Δ* and *pex11Δmdm12Δ* double mutants. For each strain, the number of peroxisomes was counted from images of two counts of at least 100 non-budding cells from two independent experiments. On each graph, the average number (± SD) of peroxisomes is indicated. (*) indicates that the difference in average number of peroxisomes per cell is significant relative to the reference single mutant strains, after statistical analyses (Student’s *t* test, *p-values* < 0.005).

It has been reported that the peroxin Pex11 and ERMES subunit Mdm34 interact and may serve as a peroxisome-mitochondria tether in glucose growing condition [4]. Therefore, we investigated the contribution of Pex11 to the high peroxisome abundance observed in *mdm12Δ* and *mdm10Δ* cells (Fig 4). In glucose growing cells, deletion of *PEX11* in a wild-type context had no influence on the peroxisome content. On the contrary, a *PEX11* deletion in *mdm12Δ* cells clearly decreased the peroxisome population to a wild-type level. A decrease of peroxisome abundance was also observed in *mdm10Δ* cells deleted for *PEX11* but the effect was less pronounced than in *mdm12Δ* cells (Fig 4). The presence of the Pex11 protein is thus required to maintain a high number of peroxisomes in *mdm12Δ* cells.

### Overexpression of *MMM1* rescues the peroxisome defect of *mdm12*Δ cells

**Fig 5:**
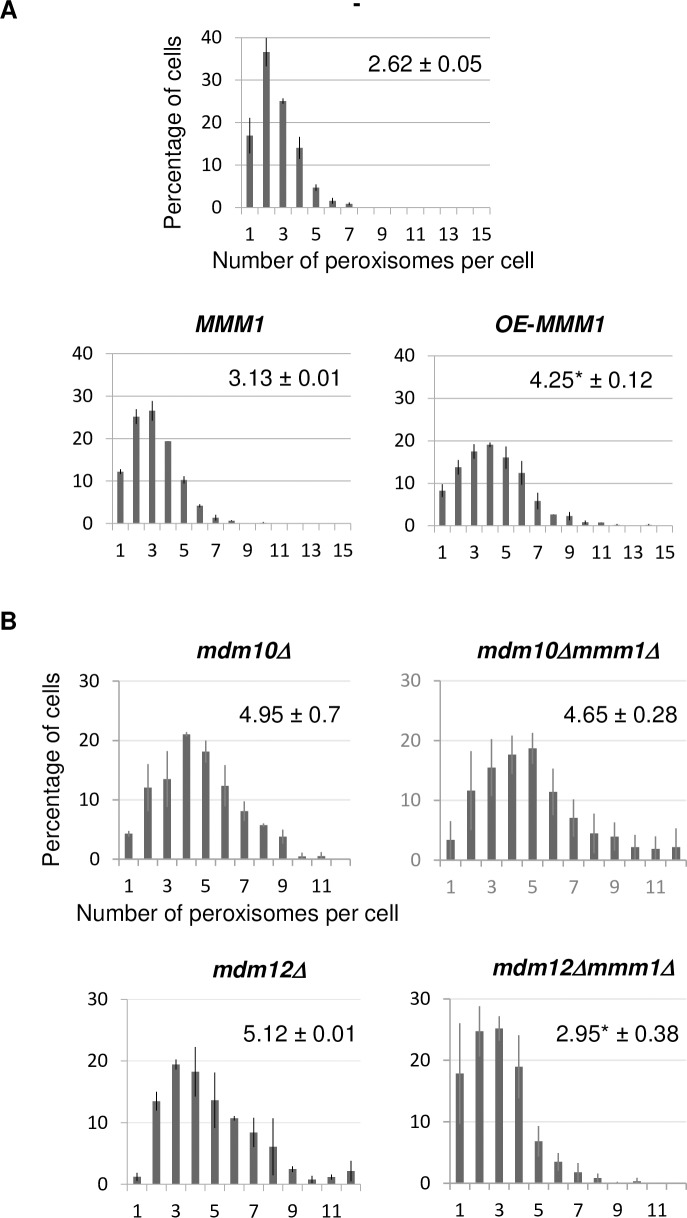
Mmm1 overproduction leads to increased peroxisome population in wild-type cells while *MMM1* deletion restores wild-type level of peroxisomes in *mdm12Δ* cells. (A) Percentage of cells for a given number of peroxisomes per cell is shown for the wild-type strain expressing or not (-) *MMM1* from its own promoter (*MMM1*) or from the strong *PGK1* promoter (OE-*MMM1*). (*) indicates that the difference in average number of peroxisomes per cell is significant relative to the reference strains, after statistical analyses (Student’s *t* test, *p-values* < 0.005). (B) Percentage of cells for a given number of peroxisomes per cell in the single *mdm10Δ* and *mdm12Δ* mutants or in the double *mdm10Δmmm1Δ* and *mdm12Δmmm1Δ* mutants. For each strain, the number of peroxisomes was counted from images of two counts of at least 100 non-budding cells from two independent experiments. On each graph, the average number (± SD) of peroxisomes is indicated. (*) indicates that the difference in average number of peroxisomes per cell is significant relative to the single mutant strains, after statistical analyses (Student’s *t* test, *p-values* < 0.005).

Contrary to the absence of Mdm10 or Mdm12, absence of the Mmm1 ERMES subunit does not increase the cellular peroxisome population. Previous studies have demonstrated that Mmm1 interacts with Mdm12 and that Mdm12 is required for Mmm1 association with mitochondria [8,19]. In addition, an Mmm1-Mdm12 complex has been shown to function as a minimal unit able to mediate *in vitro* lipid transfer between membranes [24]. We therefore addressed the effect of an imbalanced level of these two interacting proteins by first examining peroxisome population in wild-type cells that overproduced Mmm1. When Mmm1 was expressed from its own promoter on a high copy number plasmid, the average number of peroxisomes per cell was moderately increased in wild-type cells grown in glucose (Fig 5A, Table 2). When Mmm1 expression was driven from the strong *PGK1* promoter on a high copy number plasmid, peroxisome population was more strongly increased in wild-type cells (Fig 5A, Table 2). Overproduction of Mmm1 in the *mdm12Δ* context similarly increased peroxisome abundance in cells lacking the Mdm12 ERMES subunit (Table 2).

**Table 2:** Average numbers (± SD) of peroxisomes in cells overexpressing *MMM1*.

strain	Mean ± SD
BY4741 + pFL44	2.62 ± 0.05
BY4741 + pFL44-*MMM*1	3.13 ± 0.01
BY4741 + OE-*MMM1*	4.25* ± 0.12
*mdm12Δ* + pFL44	4.75 ± 0.1
*mdm12Δ* + pFL44-*MMM1*	5.70 ± 0.3
*mdm12Δ* + OE-*MMM1*	6.55* ± 0.2

Average numbers of peroxisomes per cell observed in cells overexpressing *MMM1* from its own promoter (pFL44-*MMM1*) or from the strong *PGK1* promoter (OE-*MMM1*) on multicopy plasmids in the wild-type and *mdm12Δ* cells (BY4741 genetic background). Cells were grown on glucose and the average number of peroxisomes presented as mean ± SD. Student’s *t* test, (*) *p-values* < 0.05. For each strain, the number of peroxisomes was counted from images of two counts of at least 100 non-budding cells from three independent experiments.

To assess whether the Mmm1 protein participates in peroxisome population increase in strains lacking Mdm12 or Mdm10, we evaluated the number of cellular peroxisomes in the double deleted strains *mdm10Δmmm1Δ* and *mdm12Δmmm1Δ*. Interestingly, we found that peroxisome abundance is re-established to a wild-type level in *mdm12Δ* cells lacking *MMM1* whereas absence of *MMM1* did not change peroxisome abundance in *mdm10Δmmm1Δ* cells compared to *mdm10Δ* cells (Fig 5B). Mmm1 is thus required to maintain an elevated peroxisome population in cells deleted for *MDM12* whereas it is dispensable for cells deleted for *MDM10*.

### Peroxisome turnover is not critically impaired in cells lacking Mdm12

**Fig 6:**
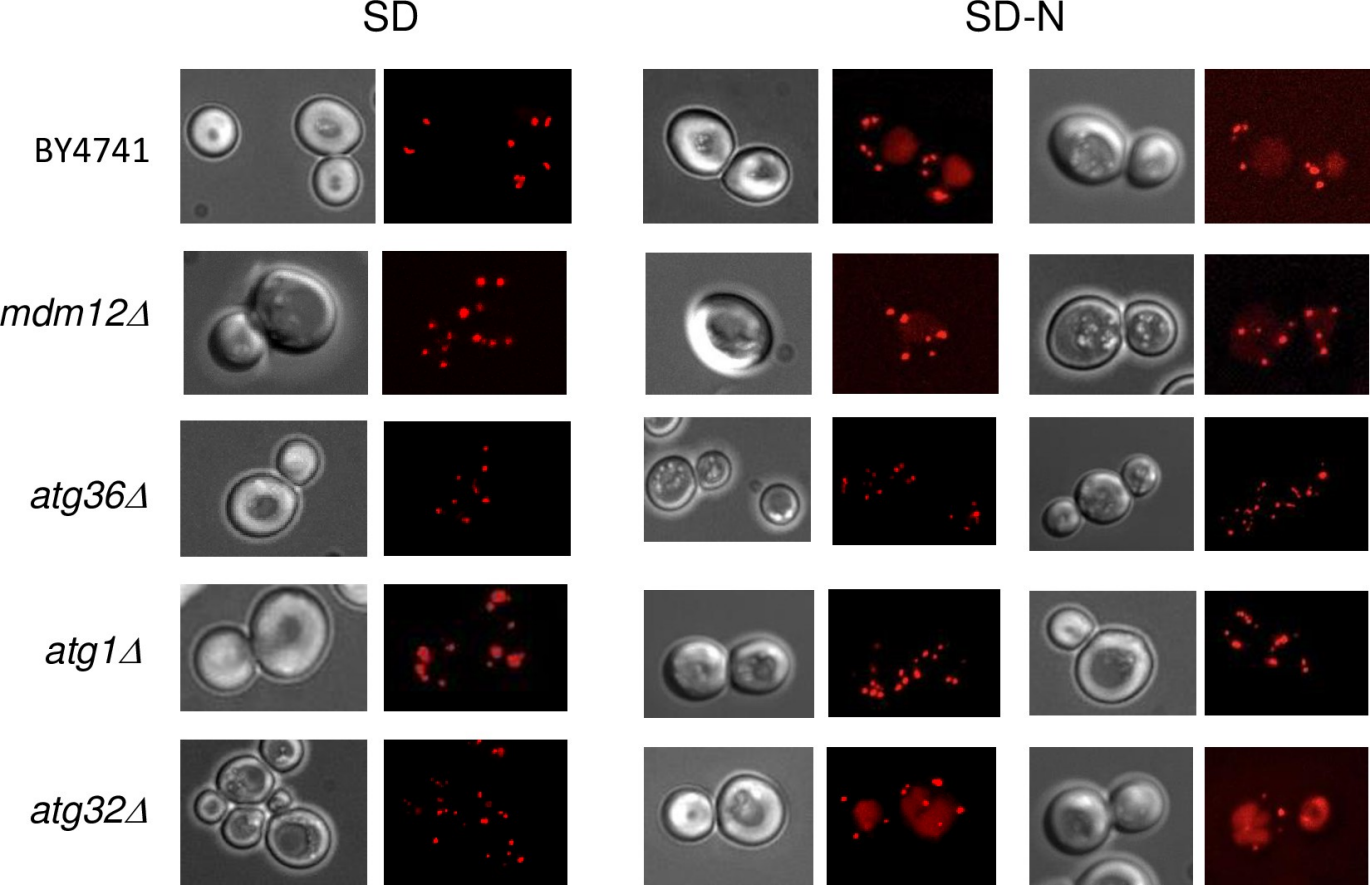
Pexophagy is not critically impaired in *mdm12Δ* cells. Wild-type (BY4741), *mdm12Δ*, *atg36Δ*, *atg1Δ* and *atg32Δ* cells expressing the Ds.Red-SKL protein targeted to the peroxisomal matrix were analyzed by fluorescence microscopy. Cells were grown in synthetic glucose (SD) or in starvation medium (glucose medium lacking nitrogen (SD-N)) for 22 hours. Typical views of the different strains are shown.

Peroxisomal abundance is regulated by organelle formation and organelle degradation [41,42]. The increased number of peroxisomes observed in cells lacking Mdm12 could be due to peroxisome accumulation if the peroxisome degradation process (*e*.*g*. pexophagy) is impaired. To investigate a possible function of Mdm12 in pexophagy, we used microscopy-based pexophagy assays that are based on the fate of Ds.Red-PTS1 labeled peroxisomes in cells grown in a starvation medium (SD-N) for 22 hours (see Materials and Methods). In this growing condition, the appearance of a vacuolar Ds.Red signal was observed in wild-type cells and is indicative of pexophagy (Fig 6). Peroxisomes remained intact and no vacuolar staining was observed in cells lacking the autophagic factors Atg1 and Atg36 (Fig 6) as previously described [42]. While cells lacking *MDM12* showed a vacuolar staining, this staining intensity was lighter than in wild-type or *atg32Δ* cells (*ATG32* is a mitophagy gene) and *mdm12Δ* peroxisome number stayed also important compared to wild-type and *atg32Δ* cells (Fig 6). These findings indicate that pexophagy is not critically impaired in cells lacking Mdm12 but do not exclude a possible slowdown of the pexophagy process.

## Discussion

One cellular response to mitochondrial dysfunction, is peroxisome proliferation by division of existing peroxisomes [29,35]. This process may relate to the finding that the loss of oxidative phosphorylation reconfigures cellular metabolism to increase supplies such as acetyl-CoA from peroxisomal activities. This merely takes place by activating a mitochondria-to-nucleus signaling pathway called the transcriptional retrograde response pathway (RTG; [33]). However, only a subset of mutant strains harboring mitochondrial dysfunction, responds by enhancing peroxisome enzyme amounts and/or by multiplying the number of peroxisomes. One recent study highlighted the involvement of the ERMES complex in peroxisome biogenesis [3]. The authors reported that, with the exception of *MMM1*, single deletion of the three other ERMES genes (*MDM34*, *MDM10* and *MDM12*) led to aberrant peroxisomes in cells observed at stationary phase. Because morphology and number of peroxisomes are highly dependent on used carbon sources and growth phases, we carefully revisited the effect of ERMES gene deletion on mature peroxisome abundance (*e*.*g*., able to import matrix protein) during logarithmic growth in glucose medium. In this condition, we found that only the absence of Mdm10 or Mdm12 leads to a higher number of mature peroxisomes when compared to a wild-type level. Although the deletion of any one gene of the ERMES complex can cause ERMES complex disassembly [8] and can lead to an alteration of the mitochondrial network and sometime loss of the mitochondrial genome (S1 Fig), the defect we observed in peroxisome abundance depends on which ERMES subunit is affected. Therefore, peroxisome proliferation in *mdm10Δ* or *mdm12Δ* deleted strains cannot be solely the consequence of mtDNA instability, respiratory dysfunction, mitochondrial morphology alteration or absence of ERMES contact sites. It indicates that it is not the function of the ERMES complex as a static tether and/or phospholipid exchanger that is involved in the proliferation of peroxisomes but rather an additional unknown role of some ERMES subunits.

It was reported that the absence of ERMES-mediated ER-mitochondria contact sites can be compensated by vacuole-mitochondria membrane contacts [25–27] for which, Mcp1 and Vps13 act as functional effectors [27]. While overexpression of *MCP1* rescues the respiratory growth defect generated by the absence of *MDM10* or *MDM12* and that dominant mutant alleles of *VPS13* alleviate *mdm10*Δ growth defect, none has an effect on peroxisome number alteration in *mdm10*Δ or *mdm12*Δ cells. These results strongly suggest that ERMES compensation through mitochondria-vacuole contact sites plays no role in high peroxisome abundance. Of note, functional peroxisomes were shown as not necessary to bypass ERMES functions [27].

What physiological role peroxisome increase could play in *mdm10Δ* and *mdm12Δ* strains? Along with mitochondria and the RE, peroxisomes house many cellular redox reactions that generate H2O2 and other reactive oxygen species (ROS), and contribute to ROS homeostasis [43]. Some evidence suggests that proliferation of peroxisomes is governed by ROS [44] and peroxisome proliferation could be a mechanism of protection against oxidative stress. However, all ERMES deleted strains we worked with did not display a resistance (or hypersensitivity) to H2O2 when grown in its presence at a concentration of 2 or 3 mM H2O2 (data not shown). Moreover, growth of the *mdm12Δmmm1Δ* cells, that contains wild-type level of peroxisomes, was found equivalent to that of *mdm12Δ* cells in the presence of H2O2 (data not shown), indicating that the high abundance of peroxisomes in *mdm12Δ* cells cannot simply be associated to a modified H2O2 sensitivity. Even if this deserves further investigations, it is unlikely that the increase in peroxisome number in *mdm10*Δ or *mdm12*Δ strains was a physiological response to an unbalanced ROS homeostasis.

### Different mechanisms sustain peroxisome proliferation in *mdm10Δ* and *mdm12Δ* cells

Our data strongly suggest that the peroxisome defect observed in the *mdm10Δ* and *mdm12Δ* strains may arise by different mechanisms. Whereas peroxisome alteration of *mdm12Δ* cells can be suppressed by different ways (absence of Mmm1 or absence of Pex11), none of those was able to restore the peroxisome defect of *mdm10Δ* cells. That defects associated to *MDM10* deletion are too drastic for the cell and cannot be restored is unlikely since several suppressors of *mdm10Δ* growth or mitochondria morphology defects have been previously identified [45,46] and overexpression of *MCP1* or *MCP2* suppresses the growth defect associated to the *mdm10Δ* strains we worked with. In addition to be an ERMES subunit, Mdm10 is also a subunit of the SAM complex that is involved in the import of β-barrel proteins of the mitochondrial outer membrane [14]. Noteworthy, *mdm10Δ* defect in assembly of mitochondrial outer membrane protein assembly was not rescued by *MCP1* or *MCP2* overexpression [40]. We cannot rule out that in *mdm10Δ* cells, alteration of mitochondrial outer membrane protein assembly might be responsible for a particular mitochondrial dysfunction that causes abnormal peroxisome proliferation. Loss of Mdm10 also led to a change in lipid composition of the mitochondria that was reported as being only partially restored by *MCP1* or *MCP2* [40]. That an imbalance in mitochondrial membrane lipid composition could be responsible for peroxisome proliferation in *mdm10Δ* cells is also an open question that deserves further investigation.

### Mdm12/Mmm1 interaction as an important player for peroxisome abundance

Our findings suggest that Mdm12 may play a specific role in maintaining a correct population of peroxisomes under condition of constitutive peroxisome division. Kawano et al. recently showed that the Mmm1–Mdm12 complex is central to the lipid transfer function of ERMES and functions as a minimal unit mediating lipid transfer between liposomes [24]. Our results showed that deregulation of peroxisome abundance can only be observed when the Mmm1/Mdm12 stoichiometry is changed either because the Mdm12 protein is absent and the Mmm1/Mdm12 complex cannot be formed, or because Mmm1 is overproduced and free ER-resident Mmm1 protein may accumulate. If the primary defect of Mdm12 loss was the release of free Mmm1 into the ER then overexpression of Mmm1 in wild-type cells would recapitulate the effect of Mdm12 loss and lead to an increase of peroxisome abundance. This is indeed the case (Fig 5A). We also found that the absence of Mmm1 abolished the increased abundance of peroxisomes in cells lacking Mdm12 (*mdm12Δmmm1Δ* cells, Fig 5B). Thus, the deregulation of the peroxisome proliferation observed in cells devoid of Mdm12, requires Mmm1. It could be hypothesized that in addition to its structural role in maintaining ER-mitochondria contact sites and its lipid exchange function in ERMES complex, the Mdm12 protein holds back Mmm1 activity on peroxisome proliferation. If this hypothesis is correct, Mdm12 and/or Mmm1 expression, as well as their steady state level might be tightly controlled. Interestingly, Mdm12 and Mdm34 have been shown to undergo multi-monoubiquitination and short-chain K63 polyubiquitination that involves the E3 ubiquitin-protein ligase Rsp5 [47]. Evaluating whether ubiquitination status of Mdm12 could modulate its interaction with Mmm1 and/or Mdm12/Mmm1 activity on peroxisome proliferation is an interesting question to address.

### Peroxisome proliferation in *mdm12Δ* cells

Several hypotheses can be drawn to explain the increased number of peroxisomes i) biogenesis of peroxisomes is cranked up, ii) peroxisome division is exacerbated or iii) peroxisome turnover is ineffective. To examine the latter point, presence of a pexophagy defect was investigated in cells devoid of Mdm12. Pexophagy (*e*.*g*., selective degradation of peroxisomes by autophagy) was not critically impaired in *mdm12Δ* cells (Fig 6), yet, we cannot exclude that peroxisome turnover was slowed down. Interestingly, efficient degradation of mitochondria by mitophagy (selective degradation of mitochondria by autophagy) was recently shown to require (i) the ERMES complex as a tether that allows lipid flux from the ER to the growing phagosome [18], and (ii) the Rsp5-mediated ubiquitination of Mdm12 and Mdm34 [47]. Taking into account that Mmm1 was here identified as an important player for peroxisome abundance, we can speculate that an excess of free Mmm1 protein or an alteration of the Mdm12/Mmm1 interaction due to Mdm12 ubiquitination, could modify the complex processes of general and selective autophagy. Of note, peroxisomes marked for degradation were shown to often localize in close vicinity to mitochondria [48]. Whether degradation of mitochondria and peroxisomes are linked to (or both initiated at) ER-mitochondria contact sites is an interesting question to address. Nevertheless, further investigations are required to determine whether peroxisome proliferation that occurs in *mdm12Δ* cells or in cells overexpressing *MMM1*, can be the result of an alteration in pexophagy.

ERMES proteins have been implicated in a large variety of cellular functions as mtDNA replication [16], mitochondrial import [14] , lipid and calcium exchange between the ER and mitochondria [8,20], mitochondria polarized transport by attachment of mitochondria to the actin cytoskeleton [19], peroxisome biogenesis [3,4] and mitophagy [18]. Our findings add new clues on the important physiological role played by three components of ERMES contact sites. ERMES represents a major site of communication/signalization between mitochondria, the ER and peroxisomes that potentially enable a dynamic three membrane junction, coordinating various aspects of the cell physiology as previously suggested by other groups [2,49].

## Materials and methods

### Yeast strains and growth conditions

Yeast strains were grown in standard rich medium with either glucose (YPD, 1% bactopeptone, 1% yeast extract, and 2% glucose) or glycerol (YPG, 1% bactopeptone, 1% yeast extract and 2% glycerol), or synthetic medium with glucose (SD, 0.67% yeast nitrogen base without amino acids and 2% glucose). Oleate induction medium (0.67% yeast nitrogen base without amino acids, 0.1% glucose, 0.1% oleate, 0.05% Tween 40, and 0.1% yeast extract, pH 6.0) was used for peroxisome proliferation experiments and SD-N (2% glucose, 0.17% yeast nitrogen base without ammonium sulfate) was used as starvation medium for the pexophagy assays. Whenever necessary, media were supplemented with the appropriate nutritional requirements according to the strains. All media were supplemented with 2% bacto agar (Difco) for solid media.

Yeast cultures were grown at 28°C. For oleate induction, cells were grown to mid log-phase (OD_600_ = 2) in glucose and then shifted to oleate for 22 hours. For drop dilution assays, cells were cultured to an optical density (600nm) of approximately 1.0 and diluted in five-fold increment followed by spotting 2.5 μl of each cell suspension on different solid medium.

Standard genetic techniques were applied for the growth and manipulation of *Saccharomyces cerevisiae* cells. The strains used in this work are listed in Table 3. Most of them were isogenic to BY4741 (*MAT*a, *his3-Δ1leu2-Δ0 met15-Δ0 ura3-Δ0*) obtained from the EUROSCARF consortium. Double mutants were generated by deleting complete open reading frames by homologous recombination and insertion of the *HIS3*MX6 cassette amplified from the plasmid pFA6a-HIS3MX6 [50] and gene specific primers. Primers are available upon request. All deletion strains were confirmed by genome-based PCR with specific primers. Transformation of yeast strains was performed by the Lithium acetate method.

**Table 3:** Strains used in the study.

Strains	Genotypes	References
BY4741	*a*, *his3-Δ1 leu2-Δ0 met15-Δ0 ura3-Δ0*	Euroscarf
*mmm1Δ*	*a*, *his3-Δ1 leu2-Δ0 met15-Δ0 ura3-Δ0 Δmmm1*::*MX6*	Euroscarf
*mdm34Δ*	*a*, *his3-Δ1 leu2-Δ0 met15-Δ0 ura3-Δ0 Δmdm34*::*MX6*	Euroscarf
*mdm10Δ*	*a*, *his3-Δ1 leu2-Δ0 met15-Δ0 ura3-Δ0 Δmdm10*::*MX6*	Euroscarf
*mdm12Δ*	*a*, *his3-Δ1 leu2-Δ0 met15-Δ0 ura3-Δ0 Δmdm12*::*MX6*	Euroscarf
*pex11Δ*	*a*, *his3-Δ1 leu2-Δ0 met15-Δ0 ura3-Δ0 Δpex11*::*MX6*	Euroscarf
*mdm10Δpex11Δ*	*a*, *his3-Δ1 leu2-Δ0 met15-Δ0 ura3-Δ0 Δpex11*::*MX6 Δmdm10*::*HIS3*	*this study*
*mdm12Δpex11Δ*	*a*, *his3-Δ1 leu2-Δ0 met15-Δ0 ura3-Δ0 Δpex11*::*MX6 Δmdm12*::*HIS3*	*this study*
*mdm10Δmmm1Δ*	*a*, *his3-Δ1 leu2-Δ0 met15-Δ0 ura3-Δ0 Δmmm1*::*MX6 Δmdm10*::*HIS3*	*this study*
*mdm12Δmmm1Δ*	*a*, *his3-Δ1 leu2-Δ0 met15-Δ0 ura3-Δ0 Δmmm1*::*MX6 Δmdm12*::*HIS3*	*this study*
*atg36Δ*	*a*, *his3-Δ1 leu2-Δ0 met15-Δ0 ura3-Δ0 Δatg36*::*MX6*	Euroscarf
*atg1Δ*	*a*, *his3-Δ1 leu2-Δ0 met15-Δ0 ura3-Δ0 Δatg1*::*MX6*	Euroscarf
*atg32Δ*	*a*, *his3-Δ1 leu2-Δ0 met15-Δ0 ura3-Δ0 Δatg32*::*MX6*	Euroscarf
W303-1B	alpha, *his3-11*,*15 leu2-3*,*112 trp1-1 ura3-1 ade2-1 can1-100*	Rothstein R
Spores from crosses BY4741 x W303-1B		
*mmm1Δ*	*his leu ura Δmmm1*::*MX6*	*this study*
*mdm34Δ*	*his leu trp1-1 ura Δmdm34*::*MX6*	*this study*
*mdm10Δ*	*his leu ura Δmdm10*::*MX6*	*this study*
*mdm12Δ*	*his leu trp1-1 ura Δmdm12*::*MX6*	*this study*

### Recombinant DNA techniques

The *MDM12* gene with its promoter and terminator sequences was amplified by PCR, sequenced and cloned into the high copy plasmid pFL44. For constitutive overproduction of the Mmm1 protein, the *MMM1* gene with or without its promoter region was amplified by PCR and cloned into pFL44 and into the high copy plasmid BFG1 that contains the strong *PGK1* promoter sequence. For overexpression of *MCP1* and *MCP2*, the coding sequences were amplified from pYX142-*MCP1* and the pYX142-*MCP2* generously obtained from K.S. Dimmer (Tuebingen, Germany; [40]) and cloned into the pVT100U (*ADH1* promoter) and BFG1 (*PGK1* promoter) multicopy plasmids, respectively. Plasmids bearing the wild-type *VPS13* gene (*VPS13*), the (D716H) mutant allele or the (L1627S) mutant were kind gifts from B. Kornmann (Zürich, Switzerland). Plasmids used to examine the cellular peroxisome number were pUG34Ds.Red-SKL(*HIS3*) or pUG34L-Ds.Red-SKL(*LEU2*) or pEH012-GFP-SKL (*URA3*) in which, Ds.Red or GFP is fused to the PTS1 peroxisomal targeting signal (SKL). The *LEU2* gene was inserted into the unique BglII sequence of pUG34Ds.Red-SKL(*HIS3*) to create the pUG34L-Ds.Red-SKL(*LEU2*) vector. The oligonucleotide sequences used for all these constructions are available upon request.

### Fluorescence microscopy and image processing

To visualize peroxisomes, yeast strains were transformed with pUG34Ds.Red-SKL*(HIS3*) or pUG34L-Ds.Red-SKL(*LEU2*) or pEH012-GFP-SKL (*URA3*). Cells were grown in glucose minimum medium with all the amino acids required to an OD_600_ around 2 (except for the pexophagy assay), pelleted and wash two times with PBS. For quantitative determination of the number of fluorescent spots per cell, counting was performed in single cells. In each quantification experiment, two counts of at least 100 non-budding cells were recorded and the experiment independently repeated. Means (± SD) presented are the means of two averages number of peroxisomes per cell calculated in at least two independent experiments.

For visualization of mitochondria, yeast cells were transformed with a vector harboring the mitochondrial targeting sequence of the subunit 9 of Fo-ATPase of *Neurospora crassa* fused to GFP or RFP [51].

Microscopy images were acquired with a DMIRE2 microscope (Leica, Deerfield, IL). Filters for GFP (450/490 nm excitation and 500/550 nm emission) and TxRED (542/582 nm excitation and 604/644 nm emission) were used. Images were captured using a CCD camera (Roper Scientific, Tucson, AZ). Metamorph software (Universal Imaging, West Chester, PA) was used to deconvolute *Z*-series and treat the images.

## Supporting information

S1 Fig Mitochondrial phenotypes of ERMES mutant strains. (A) Mitochondria of ERMES mutants constructed in the BY4741 or mixed BY/W303 genetic backgrounds were visualized with the GFP or RFP fluorescent proteins addressed to mitochondria (mtGFP and mtRFP). The mitochondrial genome was stained by DAPI or visualized with the mitochondrial DNA-binding protein Abf2 fused to GFP (Abf2-GFP). (B) Growth of drop serial dilutions of ERMES mutants, isogenic to BY4741 or to a mixed BY/W303 genetic context. Cells were grown on fermentative (YPD) or respiratory (YPG) medium.
(PDF)

S2 Fig Peroxisome content in rho+ and rho0 BY4741 cells. Wild-type (BY4741 rho+) strain and its derivative rho0 (absence of mtDNA, BY4741 rho0) both expressing a Ds.Red protein targeted to the peroxisomal matrix (Ds.Red-SKL), were stained by DAPI and analyzed by fluorescence microscopy. Typical views of the two strains are shown.
(PDF)
