# Supplementary figures and images for: Contribution of ERMES subunits to mature peroxisome abundance
Source: PLoS One. 2019 Mar 25;14(3):e0214287. doi: 10.1371/journal.pone.0214287 (PMC6433259; doi:10.1371/journal.pone.0214287)

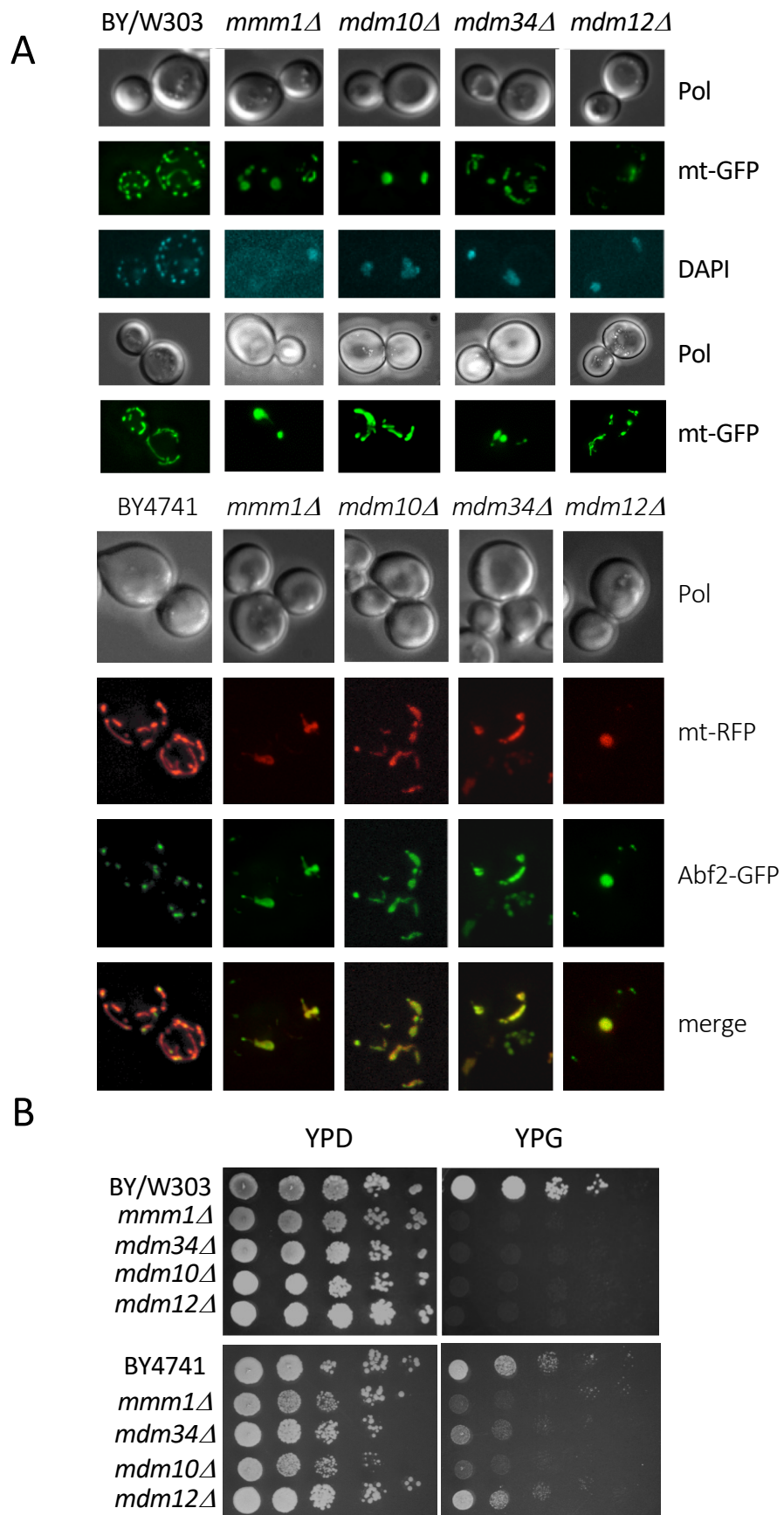

Figure S1: Mitochondrial phenotypes of ERMES mutant strains.

Supplement: S1 Fig — (A) Mitochondria of ERMES mutants constructed in the BY4741 or mixed BY/W303 genetic backgrounds were visualized with the GFP or RFP fluorescent proteins addressed to mitochondria (mtGFP and mtRFP). The mitochondrial genome was stained by DAPI or visualized with the mitochondrial DNA-binding protein Abf2 fused to GFP (Abf2-GFP). (B) Growth of drop serial dilutions of ERMES mutants, isogenic to BY4741 or to a mixed BY/W303 genetic context. Cells were grown on fermentative (YPD) or respiratory (YPG) medium. (PDF) [file pone.0214287.s001.pdf]

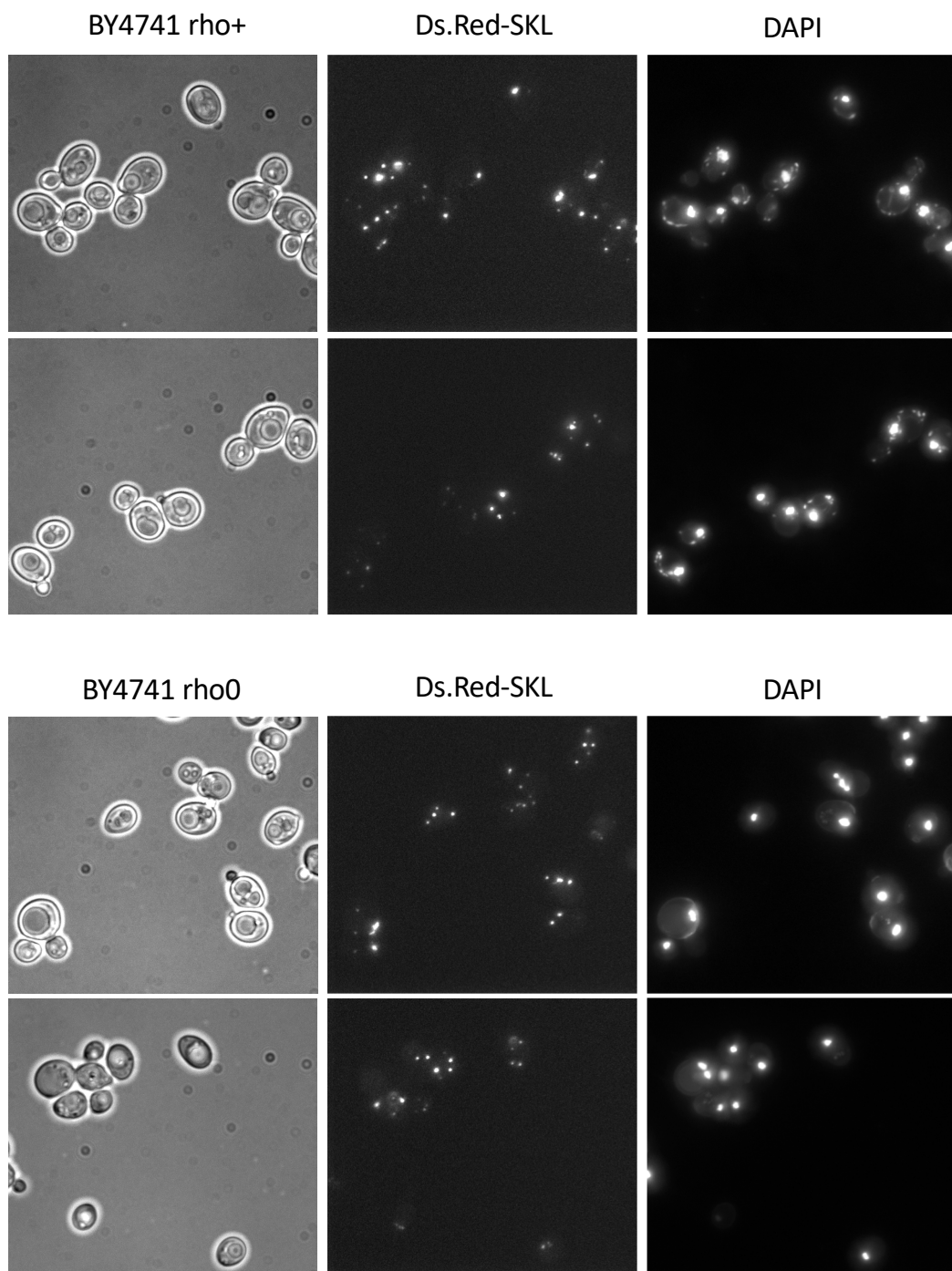

Figure S2: Peroxisome content in rho+ and rho0 BY4741 cells

Supplement: S2 Fig — Wild-type (BY4741 rho+) strain and its derivative rho0 (absence of mtDNA, BY4741 rho0) both expressing a Ds.Red protein targeted to the peroxisomal matrix (Ds.Red-SKL), were stained by DAPI and analyzed by fluorescence microscopy. Typical views of the two strains are shown. (PDF) [file pone.0214287.s002.pdf]
